# Long-term aspartame and saccharin intakes are related to greater volumes of visceral, intermuscular, and subcutaneous adipose tissue: the CARDIA study

**DOI:** 10.1038/s41366-023-01336-y

**Published:** 2023-07-13

**Authors:** Brian T. Steffen, David R. Jacobs, So-Yun Yi, Simon J. Lees, James M. Shikany, James G. Terry, Cora E. Lewis, John J. Carr, Xia Zhou, Lyn M. Steffen

**Affiliations:** 1 Division of Computational Health Sciences, Department of Surgery, School of Medicine, University of Minnesota School of Medicine, Minneapolis, MN USA; 2 Division of Epidemiology and Community Health, School of Public Health, University of Minnesota, Minneapolis, MN USA; 3 Medical Sciences Division, Northern Ontario School of Medicine University, Thunder Bay, ON Canada; 4 Division of Preventive Medicine, Heersink School of Medicine, University of Alabama at Birmingham, Birmingham, AL USA; 5 Department of Radiology and Vanderbilt Translational and Clinical Cardiovascular Research Center (VTRACC), Vanderbilt University Medical Center, Nashville, TN USA; 6 Department of Epidemiology, School of Public Health, University of Alabama at Birmingham, Birmingham, AL USA

**Keywords:** Epidemiology, Risk factors

## Abstract

**Background:**

Artificial sweetener (ArtSw) intakes have been previously associated with higher BMI in observational studies and may promote visceral and skeletal muscle adipose tissue (AT) accumulation. This study aimed to determine whether habitual, long-term ArtSw or diet beverage intakes are related to greater AT depot volumes and anthropometry-related outcomes.

**Methods:**

A validated diet history questionnaire was administered at baseline, year 7, and year 20 examinations in 3088 men and women enrolled in the Coronary Artery Risk Development in Young Adults cohort (CARDIA), mean age of 25.2 years and mean BMI of 24.5 kg/m^2^ at baseline. Volumes of visceral (VAT), intermuscular (IMAT), and subcutaneous adipose tissue (SAT) were assessed by computed tomography at year 25. Linear regression evaluated associations of aspartame, saccharin, sucralose, total ArtSw, and diet beverage intakes with AT volumes, anthropometric measures, and 25-year change in anthropometry. Cox regression estimated associations of ArtSw with obesity incidence. Adjustments were made for demographic and lifestyle factors, total energy intake, and the 2015 healthy eating index.

**Results:**

Total ArtSw, aspartame, saccharin, and diet beverage intakes were positively associated with VAT, SAT, and IMAT volumes (all *p*_trend_ ≤ 0.001), but no associations were observed for sucralose intake (all *p*_trend_ > 0.05). In addition, total ArtSw, saccharin, aspartame, and diet beverage intakes were associated with greater body mass index, body weight, waist circumference, and their increases over a 25-year period. Except for saccharin (*p*_trend_ = 0.13), ArtSw, including diet soda, was associated with greater risks of incident obesity over a median 17.5-year follow-up (all *p*_trend_ < 0.05).

**Conclusions:**

Results suggest that long-term intakes of aspartame, saccharin, or diet soda may increase AT deposition and risk of incident obesity independent of diet quality or caloric intake. Coupled with previous evidence, alternatives to national recommendations to replace added sugar with ArtSw should be considered since both may have health consequences.

## Background

The American Heart and Diabetes Associations have recommended replacing added sugar and sugar-sweetened beverages with ArtSw and diet beverages to help curb the continuing obesity and type 2 diabetes epidemics in the US [1]. While foods and beverages containing ArtSw provide less energy than their calorie-rich counterparts sweetened with sugar, honey, or high fructose corn syrup, evidence has emerged that ArtSws are not benign chemical compounds [2, 3]. More alarming, ArtSw may contribute to the conditions that they are intended to alleviate, including adipose tissue (AT) accumulation and obesity [3–7]—though this remains controversial.

Meta-analyses of observational studies have shown that ArtSw intake is positively related to waist circumference, body mass index (BMI), and risk of developing obesity [2, 3]. Moreover, experimental cell culture and animal models have shown that ArtSw may have direct adipogenic effects that promote fat accumulation, e.g., through hyperphagia [8] and or an increase in intestinal enzyme activity that has weight-related consequences [5]. And yet, null findings of ArtSw and adiposity outcomes have also been reported [9, 10]. Critically, no studies have captured habitual intakes of individual ArtSw over a long follow-up while controlling for diet quality *and* total caloric intake. Further research is therefore warranted to examine long-term habitual ArtSw exposures and adiposity outcomes, including anthropometry and specific AT compartment volumes. Visceral and skeletal muscle compartments are of particular interest, given their consistent associations with greater risks of incident type 2 diabetes [11–13] and clinical or subclinical coronary heart disease [14–16]—in contrast to the mixed results observed for subcutaneous AT compartment volumes [11, 17].

The present study aimed to determine whether total ArtSw and diet beverage intakes are associated with computed tomography (CT)-derived AT volumes independent of total caloric intake and diet quality. Because ArtSws are distinct chemical compounds, the components of total ArtSw defined in this study (aspartame, sucralose, and saccharin) were also examined as separate dietary exposures. We hypothesized that, among 3088 men and women enrolled in the Coronary Artery Risk Development in Young Adults (CARDIA) cohort study, greater intakes of total ArtSw, individual ArtSw, and diet beverages would be associated with greater volumes of visceral AT (VAT), intermuscular AT (IMAT), and subcutaneous AT (SAT) independent of demographic and lifestyle variables including total caloric intake and the healthy eating index. Anthropometric metrics, their changes over a 25-year period, and incident obesity were also examined to allow for comparisons with previous studies.

## Data and methods

### Study population

CARDIA is a prospective cohort study of young adults at baseline (1985–1986) that examines factors involved in coronary heart disease development during follow-up visits [18]. The study sample included 5115 Black and White women and men, aged 18–30 years at baseline, who were recruited across four US metropolitan areas: Birmingham, AL; Chicago, IL; Minneapolis, MN, and Oakland, CA. Institutional Review Boards at each field center approved CARDIA study protocols. All participants gave written informed consent for participation in each CARDIA exam.

### Data collection

Questionnaires assessed demographic characteristics (age, sex, race, and education) and lifestyle factors (physical activity, cigarette smoking, and alcohol intake) at baseline. A separate questionnaire was administered to assess physical activity [19]. Physical activity scores were calculated based on time spent in activities and weighted by estimated energy expenditures. Height and weight were measured by a stadiometer and beam balance scale, respectively. BMI was calculated as weight in kilograms divided by height in meters squared (kg/m^2^). Waist circumference was measured twice, and the average of the measurements was used in the analyses.

### Diet assessment

Trained and certified interviewers administered the CARDIA Diet History, a validated questionnaire used to assess usual dietary intake during the previous month [20] at exam years 0, 7, and 20. Participants were asked to provide details about foods and beverages, including brand name information and nutrient modification (e.g., artificially sweetened foods and beverages), frequency (per day, week, or month), and amounts consumed. The dietary data entry and analysis software, Nutrition Data System for Research (NDSR), was developed at the University of Minnesota Nutrition Coordinating Center and used to code dietary data into food and beverage groups and nutrient composition, including ArtSw (mg/d) aspartame, saccharin, and sucralose. Food and beverage groups (sv/d) consisted of fruit, fruit juice, vegetables, legumes, nuts, whole grain products, refined grain products, dairy, eggs, meat, fish and seafood, candy, coffee, tea, sugar-sweetened beverages, and diet beverages. One serving of diet beverages was considered equal to 8 fluid ounces. To evaluate diet quality, the Healthy Eating Index 2015 (HEI2015), which assessed compliance with the 2015–2020 Dietary Guidelines for Americans, was derived [21].

### CT measurement of AT volumes

At the year 25 exam, participants underwent multidetector CT to quantify abdominal muscle and AT volumes as previously described [13, 16]. AT volumes were measured from CT images covering the lower abdomen obtained without oral or intravenous contrast agents. Medical Image Processing, Analysis, and Visualization [(MIPAV); http://mipav.cit.nih.gov/index.php] software with a custom plugin was used to perform quantitative measurements of 4 paired muscle groups: psoas, paraspinous, lateral oblique, and rectus abdominis along with AT volumes.

AT volumes and muscle composition were measured within a 10 mm block of contiguous 1–1.25 mm slices. These CT slices were loaded into the MIPAV viewer, and the axial, coronal, and sagittal reformats were used to select the center of the lumbar disk space at L4–L5 for AT volumes and L3–L4 for muscles (to avoid artifacts produced by the pelvic bones encountered in some individuals that may obscure abdominal muscles at the lower L4–L5 level). Quality control and image analysis were performed at the CT Reading Center (Wake Forest University Health Sciences, Winston–Salem, NC). Inter-reader reliability was assessed by re-reads of 158 scan pairs with correlations of >0.95 and 0.99 for psoas muscle total volume and VAT, respectively.

### Statistical analysis

There were 3276 participants, 72% of the surviving cohort, who attended the year 25 examination. Exclusion criteria included participants with missing diet data (*n* = 5), implausible energy intake <600 and >6000 kcal/d for women and <800 and >8000 kcal/d for men (*n* = 32), missing CT scan measurement due to participant refusal or their weight exceeding the maximum table weight (158.8 kg) for the CT scan (*n* = 167), or other missing data (*n* = 2). A participant may have met more than one of these criteria. The resulting number of participants included in the analysis for body composition was n = 3 088, including 869 Black women, 867 White women, 590 Black men, and 762 White men. For the analysis of incident obesity, those with prevalent obesity at baseline (defined as BMI ≥ 30 kg/m^2^) were excluded, resulting in a sample size of 2745 participants. To assess bias in the exposure variables, ArtSw intake levels were compared between those who attended the year 25 exam and those who did not; no difference in ArtSw intake was evident.

SAS version 9.4 (SAS Institute Inc., Cary, NC) was used to analyze the data. SAS code is available by contacting the corresponding author. AT volume distributions were checked for normality. Intakes of ArtSw (mg/d) and diet beverages (sv/d) were represented as the average of years 0, 7, and/or 20, and quintiles were created. The correlation between ArtSw (mg/d) and diet beverage (sv/d) intakes was 0.33 (*p* < 0.001). Baseline characteristics were reported as means (±SE) and frequencies (%) for continuous and categorical variables, respectively, across quintiles of ArtSw. Quintiles of ArtSw were selected to capture any non-linear relationships between the exposures and outcomes. Changes between baseline and year 25 were computed for BMI, weight, and waist circumference. Statistical models were adjusted for factors that are known to be associated with BMI and AT outcomes and or are plausibly related to the ArtSw exposure, including age, sex, race, field center, education, height, smoking, alcohol consumption status, physical activity, energy intake, and HEI2015 score. Cox regression analysis estimated hazard ratios between ArtSw exposures with incident obesity (BMI ≥ 30 kg/m^2^) over a median 17.5-year follow-up period with the same covariate adjustments as above. The modifying influence of sex or race on the above associations were tested and found to be non-significant.

## Results

Baseline characteristics of 3 088 CARDIA participants are shown across quintiles of total baseline ArtSw intake in Table 1 and are adjusted for age, sex, race, education, and energy intake. Greater proportions of women and White participants, lower proportions of current smokers, and trends for higher levels of education (dichotomized by ≥high school), physical activity, energy intake, BMI, waist circumference, and fasting insulin were evident across successive quintiles of long-term total ArtSw intake. Characteristics are also presented across categories of the individual ArtSw aspartame (Supplementary Table 1), sucralose (Supplementary Table 2), and saccharin (Supplementary Table 3).

**Table 1:** Baseline (year 0) demographic characteristics, lifestyle, physical, and clinical characteristics of female and male CARDIA participants across quintiles of total artificial sweetener intake (mg/day)^a^ (*N* = 3088). Values are expressed as means ± SE or *n* (%).

Characteristics	Quintiles of total baseline artificial sweetener intake (mg/day)					
	1	2	3	4	5	*Trend p*-value
	(*n* = 617)	(*n* = 618)	(*n* = 618)	(*n* = 618)	(*n* = 617)	
Mean intake	23.3	38.32	54.38	81.87	200.62	
Range	(5.43–31.3)	(31.3–45.5)	(45.6–85.2)	(65.3–104.1)	(>104.1)	
*Demographics*						
Age, years	25.1 (±0.2)	25.2 (±0.2)	25.1 (±0.1)	25.1 (±0.2)	25.2 (±0.2)	0.99
Sex, women %	52.4 (±1.9)	47.8 (±1.8)	53.6 (±1.8)	58.9 (±1.9)	68.4 (±1.9)	<0.001
Race, White %	40.5 (±1.9)	48.9 (±1.9)	49.5 (±1.9)	55.7 (±1.9)	69.1 (±1.9)	<0.001
Education^b^	90.3 (±1.1)	91.1 (±1.0)	93.1 (±1.0)	93.7 (±1.1)	94.4 (±1.1)	0.004
*Lifestyle characteristics*						
Current smokers, %	30.5 (±1.8)	26.8 (±1.8)	24.9 (±1.7)	28.0 (±1.8)	24.2 (±1.8)	0.07
Alcohol status, %	15.2 (±1.4)	11.9 (±1.4)	12.6 (±1.3)	12.7 (±1.4)	13.7 (±1.4)	0.6
Physical activity score	390.1 (±11.6)	403.3 (11.0)	410.6 (±10.9)	423.7 (±11.2)	459.2 (±11.4)	<0.001
Energy intake, kcal	1514.9 (±38.9)	1880.4 (±38.6)	2232.1 (±38.6)	2686.7 (±38.7)	2813.3 (±39.0)	<0.001
HEI-2015 diet quality score	60.4 (±0.40)	61.9 (±0.38)	61.1 (±37.5)	60.6 (±38.4)	61.3 (±39.2)	0.81
*Physical and clinical characteristics*						
BMI, kg/m^2^	24.2 (±0.2)	24.1 (±0.2)	24.2 (±0.2)	24.6 (±0.2)	25.4 (±0.2)	<0.001
Waist circumference, cm	77.2 (±0.4)	76.9 (±0.4)	77.2 (±0.4)	78.0 (±0.4)	79.2 (±0.4)	<0.001
Insulin, pmol/L	70.8 (±2.1)	69.4 (±2.1)	70.8 (±2.1)	73.6 (±2.1)	80.6 (±2.1)	0.001
Glucose, mmol/L	4.55 (±0.03)	4.55 (±0.03)	4.56 (±0.02)	4.53 (±0.03)	4.56 (±0.03)	0.99

^a^Adjusted for age, sex, race, education, and energy intake.

^b^≥High School (%).

*CARDIA* coronary artery risk development in young adults, *HEI2015* healthy eating index-2015, *BMI* body mass index.

Associations of time-averaged ArtSw intake with AT depot volumes, anthropometric measures, and 25-year changes in anthropometric measures are shown in Table 2. Higher estimated ArtSw intake was related to greater AT volumes of VAT (*p*_trend_ = 0.001), SAT (*p*_trend_ < 0.001), and IMAT (*p* < 0.001). In terms of percent differences between extreme quintiles, those in the top quintile of total ArtSw intake showed 8.8% higher VAT, 14.8% higher SAT, and 13.3% higher IMAT volumes than those in the bottom quintile. Higher intakes of ArtSw were also related to greater year 25 BMI (*p*_trend_ < 0.001), body weight (*p*_trend_ < 0.001), and waist circumference (*p*_trend_ < 0.001), as well as increases in these metrics over a 25-year period (all *p*_trend_ ≤ 0.008).

**Table 2:** Adjusted mean (±SE) adipose tissue volumes and anthropometric measures at year 25 in CARDIA participants across quintiles of averaged (at Y0, 7, 20) total artificial sweetener intake (*N* = 3088).

Characteristic	Quintiles of Averaged Total Artificial Sweetener Intake, mg/day					
	1	2	3	4	5	Trend *p*-value
	(*n* = 617)	(*n* = 618)	(*n* = 618)	(*n* = 618)	(*n* = 617)	
Total ArtSw (mean)	22.5	37.3	53.9	83.4	394.9	
(range)	(6.34–30.3)	(30.4–45.3)	(45.3–63.9)	(64.1–111.3)	(>111.3)	
*Adipose outcome*						
VAT (mL)	127.8 (±2.3)	129.1 (±2.2)	134.2 (±2.2)	130.4 (±2.2)	139.0 (±2.2)	0.001
SAT (mL)	319.3 (±4.8)	323.2 (±4.6)	335.8 (±4.6)	332.3 (±4.7)	366.4 (±4.7)	<0.001
IMAT (mL)	2.25 (±0.06)	2.29 (±0.05)	2.42 (±0.05)	2.33 (±0.05)	2.55 (±0.06)	<0.001
*Anthropometry*						
BMI (kg/m^2^)	29.4 (±0.2)	29.6 (±0.2)	30.4 (±0.2)	30.4 (±0.2)	31.6 (±0.2)	<0.001
Weight (kg)	85.0 (±0.5)	85.9 (±0.5)	87.9 (±0.5)	88.2 (±0.5)	91.8 (±0.5)	<0.001
WC (cm)	93.2 (±0.5)	93.3 (±0.4)	94.6 (±0.4)	94.8 (±0.5)	97.3 (±0.5)	<0.001
*25-year change*^*a*^						
BMI change (kg/m^2^)	5.4 (±0.2)	5.6 (±0.2)	5.7 (±0.2)	5.9 (±0.2)	6.3 (±0.2)	0.003
Weight gain (kg)	15.6 (±0.6)	16.2 (±0.6)	16.5 (±0.6)	17.0 (±0.6)	18.2 (±0.6)	0.002
WC change (cm)	16.4 (±0.5)	16.5 (±0.5)	16.8 (±0.5)	17.6 (±0.5)	18.1 (±0.5)	0.008

Models adjusted for age, sex, race, field center, education, height, smoking, alcohol consumption status, physical activity, energy intake, and HEI2015 score.

^a^Change in BMI, weight, and WC was also adjusted for baseline BMI, weight, or WC as appropriate.

*CARDIA* coronary artery risk development in young adults, *ArtSw* artificial sweetener, *VAT* visceral adipose tissue, *SAT* subcutaneous adipose tissue, *IMAT* intermuscular adipose tissue, *HEI2015* healthy eating index-2015, *BMI* body mass index, *WC* waist circumference.

Associations of time-averaged aspartame intake with AT and anthropometric outcomes are shown in Table 3. Higher estimated aspartame intake was related to greater AT volumes of VAT (*p*_trend_ < 0.001), SAT (*p*_trend_ < 0.001), and IMAT (*p*_trend_ < 0.001). Those in the top quintile of aspartame intake showed 8.4% higher VAT, 11.6% higher SAT and 10.6% higher IMAT volumes than those in the bottom quintile. Greater intake of aspartame was further related to greater BMI (*p*_trend_ < 0.001), weight (*p*_trend_ < 0.001), waist circumference (*p*_trend_ < 0.001), and increases in these metrics over a 25-year follow-up period (all *p*_trend_ ≤ 0.03).

**Table 3:** Adjusted mean (±SE) adipose tissue volumes and anthropometric measures at year 25 in CARDIA participants across quintiles of averaged (at Y0, 7, 20) aspartame intake (*N* = 3088).

Characteristic	Quintiles of averaged aspartame intake, mg/day					
	1	2	3	4	5	*Trend p*-value
	(*n* = 617)	(*n* = 618)	(*n* = 618)	(*n* = 618)	(*n* = 617)	
Aspartame (mean)	3.0	4.7	6.8	13.9	106.5	
(range)	(0.8–3.9)	(3.9–5.6)	(5.6–8.4)	(8.4–28.5)	(>28.5)	
*Adipose outcome*						
VAT (mL)	127.9 (±2.3)	127.0 (±2.2)	134.4 (±2.2)	132.6 (±2.3)	138.6 (±2.2)	<0.001
SAT (mL)	322.7 (±4.9)	323.2 (±4.6)	335.0 (±4.7)	336.2 (±4.8)	360.0 (±4.7)	<0.001
IMAT (mL)	2.26 (±0.06)	2.26 (±0.05)	2.45 (±0.05)	2.38 (±0.06)	2.50 (±0.06)	<0.001
*Anthropometry*						
BMI (kg/m^2^)	29.6 (±0.2)	29.6 (±0.2)	30.4 ( ± 0.2)	30.4 ( ± 0.2)	31.5 ( ± 0.2)	<0.001
Weight (kg)	85.6 (±0.6)	85.8 (±0.5)	88.3 ( ± 0.5)	88.0 ( ± 0.6)	91.1 ( ± 0.5)	<0.001
WC (cm)	92.9 (±0.5)	93.3 (±0.4)	95.1 ( ± 0.5)	94.7 ( ± 0.5)	97.3 ( ± 0.5)	<0.001
*25-year change*^a^						
BMI change (kg/m^2^)	4.3 (±0.2)	5.8 (±0.2)	6.0 (±0.2)	5.8 (±0.2)	6.0 (±0.2)	0.03
Weight gain (kg)	15.3 (±0.6)	16.7 (±0.6)	17.4 (±0.6)	16.7 (±0.6)	17.4 (±0.6)	0.03
WC change (cm)	15.6 (±0.5)	17.0 (±0.5)	17.6 (±0.5)	16.9 (±0.5)	17.7 (±0.5)	0.01

Models adjusted for age, sex, race, field center, education, height, smoking, alcohol consumption status, physical activity, energy intake, and HEI2015 score

^a^Change in BMI, weight, and WC was also adjusted for baseline BMI, weight, or WC as appropriate.

*CARDIA* coronary artery risk development in young adults, *VAT* visceral adipose tissue, *SAT* subcutaneous adipose tissue, *IMAT* intermuscular adipose tissue, *HEI2015* healthy eating index-2015, *BMI* body mass index, *WC* waist circumference.

Analyses of saccharin intake are shown in Table 4. Saccharin was categorized by tertiles instead of quintiles due to the number of individuals with no self-reported intake (*n* = 1919). Like aspartame, greater intake of saccharin was related to greater AT volumes in visceral (*p*_trend_ = 0.001), subcutaneous (*p*_trend_ < 0.001), and intermuscular compartments (*p*_trend_ < 0.001) across tertiles of intake. Those in the top tertile of saccharin intake showed 10.3% higher VAT, 14% higher SAT, and 10% higher IMAT volumes than those in the bottom tertile. Greater intake of saccharin was additionally related to greater BMI (*p* < 0.001), weight (*p*_trend_ < 0.001), waist circumference (*p*_trend_ < 0.001) as well as 25-year increases in weight (*p*_trend_ = 0.03) and waist circumference (*p*_trend_ = 0.008) but not BMI (*p*_trend_ = 0.06).

**Table 4:** Adjusted mean (±SE) year 25 adipose tissue volumes and anthropometric measures of CARDIA participants across tertiles of averaged saccharin intake (*N* = 3088).

Characteristic	Tertiles of averaged Saccharin intake (mg/day)			Trend *p*-value
	1 (*n* = 1919)	2 (*n* = 597)	3 (*n* = 572)	
Saccharin (mean)	0	6.01	65.62	
(range)		(>0–17.1)	(>17.1)	
*Adipose outcome*				
VAT (mL)	128.3 (±1.7)	135.2 (±2.9)	141.6 (±3.1)	<0.001
SAT (mL)	323.6 (±3.7)	341.5 (±6.4)	368.8 (±6.8)	<0.001
IMAT (mL)	2.29 (±0.03)	2.22 (±0.11)	2.52 (±0.04)	<0.001
*Anthropometry*				
BMI (kg/m^2^)	29.7 (±0.2)	30.6 (±0.3)	32.0 (±0.3)	<0.001
Weight (kg)	85.9 (±0.5)	88.8 (±0.8)	92.8 (±0.9)	<0.001
WC (cm)	93.5 (±0.4)	95.0 (±0.6)	98.2 (±0.7)	<0.001
*25-year change*^a^				
BMI change (kg/m^2^)	5.7 (±0.1)	5.7 (±0.2)	6.2 (±0.2)	0.06
Weight gain (kg)	16.3 (±0.4)	16.6 (±0.6)	18.0 (±0.6)	0.03
WC change (cm)	16.7 (±0.3)	16.6 (±0.5)	18.3 (±0.4)	0.008

Models adjusted for age, sex, race, field center, education, height, smoking, alcohol consumption status, physical activity, energy intake, and HEI2015 score.

^a^Change in BMI, weight, and WC was also adjusted for baseline BMI, weight, or WC as appropriate.

*CARDIA* Coronary Artery Risk Development in Young Adults, *VAT* visceral adipose tissue, *SAT* subcutaneous adipose tissue, *IMAT* intermuscular adipose tissue, *HEI2015* healthy eating index-2015, *BMI* body mass index, *WC* waist circumference.

The results of diet beverage intake analyses are shown in Table 5. Increasing quintiles of diet beverage intake were associated with greater AT volumes of VAT (*p*_trend_ = 0.001), SAT (*p*_trend_ < 0.001), and IMAT (*p*_trend_ < 0.001) across quintiles. Those in the top quintile of diet beverage intake showed 10.4% higher VAT, 14.1% higher SAT and 14.8% higher IMAT volumes than those in the bottom quintile. Diet beverage intake was additionally related to greater BMI (*p*_trend_ < 0.001), weight (*p*_trend_ < 0.001), waist circumference (*p*_trend_ < 0.001) as well as 25-year increases in BMI (*p*_trend_ = 0.006), weight (*p*_trend_ = 0.002) and waist circumference (*p*_trend_ = 0.002).

**Table 5:** Adjusted mean (±SE) year 25 adipose tissue volumes and anthropometric measures of CARDIA participants across quintiles of averaged diet beverage intake (servings/day at years 0, 7, and 20) (*N* = 3088).

	Quintiles of averaged diet beverage consumption (servings/day)					
Characteristic	1	2	3	4	5	Trend *p*-value
	(*n* = 1009)	(*n* = 223)	(*n* = 626)	(*n* = 609)	(*n* = 621)	
Intake (mean)	0	0.03	0.19	0.63	2.33	
(range)		(0–0.07)	(0.07–0.36)	(0.36–0.99)	( ≥1.0)	
*Adipose outcome*						
VAT (mL)	128.9 (±1.8)	124.4 (±3.6)	131.7 (±2.2)	130.2 (±2.2)	142.3 (±2.3)	<0.001
SAT (mL)	321.0 (±3.8)	318.1 (±7.7)	331.9 (±4.6)	337.9 (±4.7)	366.3 (±4.8)	<0.001
IMAT (mL)	2.29 (±0.04)	2.23 (±0.09)	2.29 (±0.05)	2.37 (±0.05)	2.63 (±0.06)	<0.001
*Anthropometry*						
BMI (kg/m^2^)	29.7 (±0.2)	29.6 (±0.3)	30.0 (±0.2)	30.4 (±0.2)	31.7 (±0.2)	<0.001
Weight (kg)	85.8 (±0.5)	85.5 (±0.9)	87.0 (±0.5)	88.3 (±0.5)	92.0 (±0.6)	<0.001
WC (cm)	93.5 (±0.4)	92.7 (±0.7)	93.8 (±0.4)	95.0 (±0.5)	97.8 (±0.5)	<0.001
*25-year change*^a^						
BMI change (kg/m^2^)	5.4 (±0.2)	5.5 (±0.3)	5.8 (±0.2)	6.2 (±0.2)	6.1 (±0.2)	0.006
Weight gain (kg)	15.6 (±0.5)	15.7 (±1.0)	17.0 (±0.6)	17.9 (±0.6)	17.6 (±0.6)	0.002
WC change (cm)	16.3 (±0.4)	15.6 (±0.8)	17.0 (±0.5)	17.7 (±0.5)	17.8 (±0.5)	0.002

Models adjusted for age, sex, race, field center, education, height, smoking, alcohol consumption status, physical activity, energy intake, and HEI2015 score

^a^Change in BMI, weight, and WC was also adjusted for baseline BMI, weight, or WC as appropriate.

*CARDIA* coronary artery risk development in young adults, *VAT* visceral adipose tissue, *SAT* subcutaneous adipose tissue, *IMAT* intermuscular adipose tissue, *HEI2015* healthy eating index-2015, *BMI* body mass index, *WC* waist circumference.

The results of sucralose analyses are shown in Supplementary Table 4. Estimated sucralose intake was associated with incrementally greater BMI (*p*_trend_ = 0.004), weight (*p*_trend_ < 0.001), and waist circumference (*p*_trend_ = 0.05) across quintiles. No significant associations were observed between sucralose intake and AT volumes or changes in anthropometry over 25 years.

**Fig. 1:**
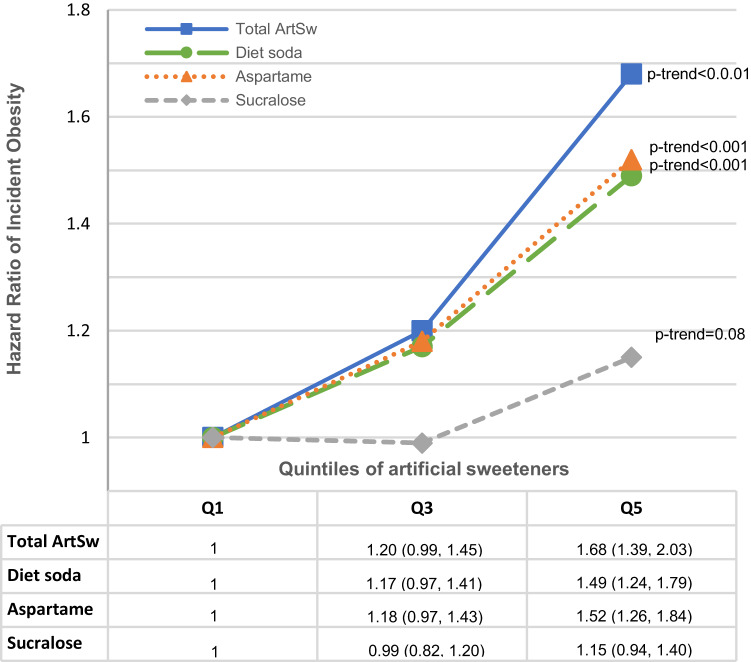
ArtSw and obesity incidence. Risk of incident obesity associated with total ArtSw, aspartame, sucralose, and diet soda intakes [hazard ratios (95% CIs)] over a median 17.5-year period among CARDIA participants (*N* = 2745, *n* events = 1142).

Risks of incident obesity associated with total ArtSw, aspartame, sucralose, and diet soda intakes over a median 17.5-year follow-up are shown in Fig. 1. Significant trends in greater risks were observed across quintiles of total ArtSw (*p*_trend_ < 0.001), aspartame (*p*_trend_ < 0.001), diet beverage (*p*_trend_ < 0.001), and sucralose intakes (*p*_trend_ = 0.018). Comparing extreme quintiles, individuals in the top quintiles of total ArtSw, aspartame, and diet beverage intakes showed 78%, 64%, and 57% greater risks for incident obesity than corresponding bottom quintiles, respectively (Fig. 1). All results are shown in Supplementary Table 5. Modest 13% and 19% greater risks of incident obesity were observed for individuals in the 2^nd^ and 3^rd^ tertiles of saccharin intake, respectively; however, the trend across tertiles was non-significant (*p*_trend_ = 0.13).

## Discussion

This is the first observational study to examine habitual intakes of individual ArtSw and their associations with adiposity-related outcomes over a long follow-up. Among CARDIA participants, higher intakes of total ArtSw and diet beverages were associated with greater VAT, SAT, and IMAT, with approximately 8–15% greater volumes for those in the top quintiles compared to those in the bottom quintiles. For individual ArtSw, associations of aspartame and saccharin intake with AT volumes were consistent with those of total ArtSw and diet beverage intake, but no associations were observed for sucralose. In agreement with previous observational studies, all ArtSw exposures were related to higher BMI, body weight, and waist circumference and, except for sucralose, were associated with increases in these anthropometric measurements over a 25-year period. Greater risks of developing obesity were found across increasing quintiles of total ArtSw, aspartame, sucralose, and diet beverage intakes.

### Previous findings from randomized controlled trials (RCTs) and cohort studies

Contrary to our findings and those of other observational studies, RCTs of ArtSw have demonstrated modest but inconsistent weight loss effects, which are likely due to controlled or reduced caloric intake among the individuals participating in these trials. A 2014 meta-analysis of 15 weight management RCTs in children and adults over a median 12-week time course showed that *replacing* sugar or sugar-sweetened beverages with ArtSw or ArtSw-containing beverages resulted in modest but statistically significant weight loss (−0.80 kg), reductions in mean BMI (−0.24 kg/m^2^), or lower fat mass (−1.10 kg) [9]. Likewise, a meta-analysis of 29 intervention studies showed that replacing sugar intake with ArtSw resulted in an approximate 1 kg reduction in body weight over a median study period of 12 weeks [10]. And yet, when ArtSw intake was compared with controls of water or no dietary change over a median 12-week study period, a 161 daily increase in caloric intake per SD of ArtSw intake was observed—though no accompanying change in weight status was apparent over the relatively brief study periods [10]. In evaluating the above evidence, it is important to consider the study designs, sample populations, and trial periods. Because many of these trials were testing ArtSw *as a replacement* for sugars and or were conducted in tandem with weight loss programs, they likely do not capture real-world ArtSw consumption in free-living adults over a period of decades.

Supporting this hypothesis, observational studies have shown that ArtSw or diet beverage intake may promote adiposity, higher BMI, and greater waist circumference. In a prospective study of 749 male and female older adults, diet beverage consumers showed greater waist circumferences and increases in waist circumference compared with those who did not drink diet beverages over a mean 9.4-year follow-up [7]—consistent with our findings. A 2014 meta-analysis of nine prospective cohort studies with 1 to 7.5-year follow-ups reported that ArtSw intake, assessed by diet beverage consumption, was related to a modest signal for greater BMI (0.03 kg/m^2^) [9]. A subsequent meta-analysis likewise reported a modest but significant correlation between ArtSw intake and BMI (β = 0.05; CI: 0.03–0.06) in two smaller observational studies [3], while a larger cohort study of 3371 participants reported that those who consumed ArtSw daily showed a mean 0.77 kg/m^2^ greater BMI compared with non-consumers over an 8-year follow-up [22].

In comparison to these previous observational studies, we found greater differences in BMI (ranging from 1 to 3.6 kg/m^2^) and waist circumference (ranging from 1.7 to 6.4 cm) across quantiles of total ArtSw, aspartame, saccharin, and diet beverage intakes averaged across three timepoints over 20 years. The non-significant trend in associations across tertiles of saccharin with AT volumes suggests that, of these, it may be the preferred ArtSw in terms of these outcomes; however, the modest but significant association between saccharin and incident obesity for individuals in the 2nd tertile [HR: 1.13 (95% CI: 1.06–1.20)] and 3rd tertile [HR: 1.19 (95% CI: 1.13–1.26)] of intake remain a concern (Supplementary Table 5).

Our findings that greater ArtSw intakes were related to the development of obesity are consistent with previous studies. Among 2 571 San Antonio Heart Study participants, twofold higher odds of developing obesity were observed in those who consumed 22 or more diet beverages per week than non-consumers, and a dose-response was observed across the five categories of diet beverage consumption [22]. More recently, it was shown that ArtSw users were at a 53% greater risk of incident obesity than non-users over a median 10-year follow-up [4]. We extend these findings to individual ArtSw, showing that aspartame intake was associated with an incrementally greater risk of incident obesity than either sucralose or saccharin (Supplementary Table 5), suggesting that the type of ArtSw is an important consideration for this outcome.

### Potential adipogenic mechanisms and Implications for future research

In both human and animal models, ArtSw has been shown to induce or associate with pathophysiological responses that may account for the significant associations with AT volumes and obesity development observed here. Among these, ArtSw has been shown to promote hyperphagia or increased appetite [8], and our results support a partial hyperphagic effect, i.e., ArtSw intake was positively associated with energy intake (Table 1). However, the relationships of aspartame and saccharin with AT volumes were independent of energy intake (Tables 2–5), suggesting that other mechanisms are mediating our primary observations.

Consistent with this hypothesis, potential aspartame- and saccharin-mediated mechanisms have been identified. In an adipocyte cell culture model, saccharin was found to induce adipogenesis in a dose-dependent manner as well as promote lipid accumulation and suppress lipolysis [6]. For aspartame, a more complex hypothesis has emerged involving its metabolism into phenylalanine and suppression of an intestinal enzyme, which then has numerous downstream consequences. In an experimental rodent model, it was found that aspartame consumption suppresses the activity of intestinal alkaline phosphatase (IAP) [5]—a well-described enzyme with numerous functions, including maintaining gut homeostasis by detoxifying lipopolysaccharide [23, 24]. Lower IAP enzymatic activity has further been proposed to increase gut permeability and reduce the inactivation of lipopolysaccharide [25], which would, in turn, precipitate endotoxemia, low-grade inflammation, and subsequent adipogenesis and AT accumulation [24, 26–30]. Taken together, these mechanisms would increase AT volumes, but their links with ArtSw intake are not yet definitive. Additional research is warranted to scrutinize the apparent adipogenic effects of saccharin and the link of aspartame with IAP activity suppression to determine whether they are relevant in human physiology.

### Strengths and limitations

In terms of strengths, the CARDIA study enrolled a large cohort of geographically diverse Black and White men and women aged 18–30 years at baseline who were followed for 25 years. Dietary intake was assessed at three time points over 20 years by the CARDIA diet history, which queried brand name information and whether foods were modified for nutrient content, including total energy, fat, and sweetener composition. This enabled us to estimate ArtSw content, type of ArtSw, and diet beverage intake. By contrast, most diet studies rely on food frequency questionnaires that do not query brand name information and therefore do not capture ArtSw content apart from diet beverages. To date, no objective biomarkers of habitual ArtSw intake have been validated for ArtSw intake [31, 32], so our approach of averaging ArtSw intake at multiple time points remains one of the more robust methods for assessing ArtSw intake. For AT outcomes, ATs and muscle composition were quantified using CT imaging, which precisely evaluates specific body AT compartment volumes. Finally, in terms of inferring causality, our results are largely consistent with a dose-response of ArtSw exposures with AT outcomes, and both longitudinal analyses of anthropometric measures and Cox regression of ArtSw as a time-varying exposure suggest temporality between exposures and outcomes.

The present analysis has limitations that must be acknowledged. First, ArtSw intake been shown to alter the microbiome and differentially affect glycemic responses [33], which may, in turn, influence adipogenesis [34–37]. We were unable to evaluate this putative effect. In addition, the potential for reverse causality is unlikely but remains possible [38]. For example, individuals with higher BMIs and AT stores may consume more energy and ArtSw-containing foods and beverages. Alternatively, these individuals may have consumed greater amounts of ArtSw to lose weight over the study period. To control for these possibilities, we adjusted for total energy intake and HEI2015 diet quality score over the study period; moreover, the long follow-up period coupled with the measurement of ArtSw intake at multiple time points also reduces the probability of reverse causality. In terms of reporting bias, self-reported dietary intake is noted for its measurement error; however, that bias would be expected to skew results toward null findings. Diet questionnaires did not capture information on the use of newer ArtSw like Stevia or Advantame, so we were unable to assess their associations with anthropometric or AT outcomes. Finally, the generalizability of our findings should be examined in other populations with and without a Western dietary pattern.

## Conclusions

This study provides novel evidence that habitual, long-term intakes of total and *individual* ArtSw are related to greater volumes of VAT, IMAT, and SAT compartments independent of total caloric intake and diet quality. We extended previous findings by showing associations of individual ArtSw intakes with anthropometric measures, their change over 25 years, and incident obesity. Given the cardiometabolic consequences of AT accumulation, particularly in visceral and intermuscular compartments, further studies are warranted to determine whether these ArtSws may be causal factors. Coupled with evidence from microbiome and experimental studies, our findings suggest that alternatives to the national recommendations to replace added sugar with ArtSw should be considered since both may have health consequences.

### Supplementary information

Supplemental Material

## Data Availability

The data analyzed for this study are available through the CARDIA Coordinating Center (coc@uab.edu) upon reasonable request. This may include the completion of a data and material distribution agreement.
