# Supplementary material for: Long-term aspartame and saccharin intakes are related to greater volumes of visceral, intermuscular, and subcutaneous adipose tissue: the CARDIA study
Source: Int J Obes (Lond). 2023 Jul 13;47(10):939–47. doi: 10.1038/s41366-023-01336-y (PMC10511315; doi:10.1038/s41366-023-01336-y)
Supplement: Supplementary file 1 — Supplemental Material [file 41366_2023_1336_MOESM1_ESM.pdf]

Supplementary Table 1. Baseline (year 0) demographic, lifestyle, physical and clinical characteristics* of female and male CARDIA participants across quintiles of aspartame sweetener intake (milligrams/day) (N=3 088).

Characteristics	Quintiles of Baseline Aspartame Intake (mg/day)					
	1 (n=617)	2 (n=618)	3 (n=618)	4 (n=618)	5 (n=617)	*Trend p-value*
Mean intake<br>Range	23.3<br>(5.43-31.3)	38.32<br>(31.3-45.5)	54.38<br>(45.6-85.2)	81.87<br>(65.3-104.1)	200.62<br>(>104.1)	
<u>Demographics</u>						
Age, years	25.1 (0.2)	25.2 (0.1)	25.2 (0.1)	25.2(0.2)	24.9 (0.2)	0.43
Sex, women %	62.0 (1.9)	56.1 (1.9)	47.8 (1.9)	53.8 (1.9)	61.7 (1.9)	0.62
Race, White	41.3 (2.0)	49.2 (1.9)	51. (1.9)	49.8 (2.0)	72.5 (1.9)	<0.001
Education†	91.6 (1.1)	90.9 (1.0)	91.4 (1.0)	94.0 (1.1)	94.8 (1.1)	0.006
<u>Lifestyle characteristics</u>						
Current smokers, %	28.2 (1.8)	26.1 (1.8)	28.4 (1.8)	26.7 (1.8)	25.3 (1.8)	0.36
Alcohol status, %	16.7 (1.4)	12.6 (13.4)	12.9 (1.4)	12.2 (1.4)	11.5 (1.4)	0.014
Physical activity score	399.6 (11.5)	398.1 (11.0)	418.2 (11.1)	441.9 (11.4)	429.1 (11.19)	0.005
Energy intake, kcal	1620.1 (40.9)	2019.7 (40.3)	2448.5 (40.5)	2835.1 (40.5)	2203.9 (41.2)	<0.001
HEI-2015 diet quality score	58.7 (0.4)	61.5 (0.4)	62.3 (0.4)	62.8 (0.4)	60.2 (0.4)	0.0004
<u>Physical and Clinical Characteristics</u>						
BMI, kg/m^2	23.9 (0.2)	23.9 (0.2)	24.6 (0.2)	24.5 (0.2)	25.7 (0.2)	<0.001
Waist circumference, cm	76.5 (0.4)	76.3 (0.4)	78.0 (0.4)	77.5 (0.4)	80.2 (0.4)	<0.001
Insulin, uU/mL	9.8 (0.3)	10.0 (0.3)	10.6 (0.3)	10.5 (0.3)	11.7 (0.3)	<0.001
Glucose, mg/100 mL	81.3 (0.5)	81.7 (0.4)	81.7 (0.5)	81.9 (0.5)	83.3 (0.5)	0.003

*Adjusted for age, sex, race, education, and energy intake
† ≥High School (%)
ArtSw=Artificial sweetener

Supplementary Table 2. Baseline (year 0) demographic, lifestyle, physical and clinical characteristics* of female and male CARDIA participants across quintiles of sucralose intake (milligrams/day) (N=3 088).

Characteristics	Quintiles of Baseline Sucralose Intake (mg/day)					
	1<br>(n=617)	2<br>(n=618)	3<br>(n=618)	4<br>(n=618)	5<br>(n=617)	*Trend* *p*-value
Mean intake	23.3	38.3	54.4	81.9	200.6	
Range	(5.43-31.3)	(31.3-45.5)	(45.6-85.2)	(65.3-104.1)	(>104.1)	
Demographics						
Age, years	25.2 (0.2)	25.3 (0.1)	25.1 (0.1)	25.0 (0.1)	25.0 (0.2)	0.19
Sex, women %	57.8 (1.9)	53.2 (1.9)	57.7 (1.8)	51.1 (1.9)	61.3 (2.0)	0.47
Race, White	56.1 (2.0)	54.8 (1.9)	57.0 (1.9)	45.5 (1.9)	50.4 (2.1)	0.003
Education†	91.2 (1.1)	92.0 (1.1)	93.4 (1.0)	93.8 (1.1)	92.2 (1.1)	0.31
Lifestyle characteristics						
Current smokers, %	30.0 (1.8)	26.0 (1.8)	27.3 (1.7)	26.2 (1.8)	25.2 (1.9)	0.14
Alcohol status, %	13.8 (1.4)	10.6 (1.4)	11.8 (1.3)	14.4 (1.4)	15.6 (1.4)	0.12
Physical activity score	412.5 (11.4)	408.8 (11.1)	424.1 (10.9)	420.7 (11.1)	420.8 (11.7)	0.47
Energy intake, kcal	1635.6 (39.3)	1842.0 (38.9)	2190.1 (38.9)	2469.9 (39.2)	2990.3 (39.3)	<0.001
HEI-2015 diet quality score	60.5 (0.38)	61.9 (0.4)	61.2 (0.4)	61.1 (0.4)	60.6 (0.4)	0.62
Physical and Clinical Characteristics						
BMI, kg/m^2	24.4 (0.2)	24.0 (0.2)	24.4 (0.2)	25.7 (0.2)	25.1 (0.2)	0.003
Waist circumference, cm	77.2 (0.4)	76.4 (0.4)	77.8(0.4)	78.2 (0.4)	78.9 (0.4)	<0.001
Insulin, uU/mL	10.2 (0.3)	10.1 (0.3)	10.3 (0.3)	10.9 (0.3)	11.2 (0.3)	0.01
Glucose, mg/100 mL	82.7 (0.5)	81.6 (0.5)	81.8 (0.4)	81.9 (0.5)	81.9 (0.5)	0.39

*Adjusted for age, sex, race, education, and energy intake
† ≥High School (%)
ArtSw=Artificial sweetener

Supplementary Table 3. Baseline (year 0) demographic, lifestyle, physical and clinical characteristics* of female and male CARDIA participants across tertiles of saccharin intake (milligrams/day) (N=3 088).

	Tertiles of Averaged Saccharin Intake (mg/day)			
	1<br>(n=1919)	2<br>(n=597)	3<br>(n=592)	*Trend p*-value
Mean intake<br>Range	0	6.0<br>(>0-17.1)	65.6<br>(>17.1)	
<u>Demographics</u>				
Age, years	25.0 (0.1)	25.6 (0.3)	25.29(0.12)	0.04
Sex, women %	49.7 (1.1)	64.3 (3.9)	67.27(1.47)	<0.001
Race, White	42.4 (1.1)	47.8 (3.9)	72.66(1.46)	<0.001
Education†	91.7 (0.6)	93.69(2.20)	93.88(8.39)	0.04
<u>Lifestyle characteristics</u>				
Current smokers, %	29.4 (1.01)	19.7 (1.9)	25.8 (1.8)	0.097
Alcohol status, %	13.6 (0.78)	15.0 (1.4)	10.4 (1.4)	0.05
Physical activity score	387.5 (6.33)	455.9 (11.6)	475.7 (11.3)	<0.001
Energy intake, kcal	2300.9 (25.13)	2118.9 (92.0)	2099.6 (35.0)	<0.001
HEI-2015 diet quality score	60.2 (0.22)	62.8 (0.8)	62.4 (0.3)	<0.001
<u>Physical and Clinical Characteristics</u>				
BMI, kg/m^2	24.1 (0.1)	24.8 (0.2)	25.7 (0.2)	<0.001
Waist circumference, cm	77.0 (0.2)	78.1 (0.4)	79.6 (0.4)	<0.001
Insulin, uU/mL	10.1 (0.2)	11.0 (0.3)	11.5 (0.3)	<0.001
Glucose, mg/100 mL	81.8 (0.3)	82.1 (0.5)	82.4 (0.5)	0.26

*Adjusted for age, sex, race, education, and energy intake
† ≥High School (%)
ArtSw=Artificial sweetener

Supplementary Table 4. Adjusted mean (SE) year 25 adipose tissue volumes and anthropometric measures of female and male CARDIA participants across quintiles of averaged sucralose intake (mg/day at years 0, 7, and 20) (N=3 088).

Characteristic	Quintiles of Averaged Sucralose Intake, mg/day					*Trend p-* value
	1 (n=617)	2 (n= 618)	3 (n= 618)	4 (n= 618)	5 (n= 617)	
Mean intake	14.1 (3.6)	22.9 (2.2)	31.5 (2.9)	45.3 (5.4)	276.7 (600.6)	
Range	(0-19.1)	(19.1-26.7)	(26.7-37.1)	(37.1-56.3)	(≥56.3)	
Adipose outcome						
VAT (mL)	131.2 (2.3)	130.0 (2.2)	131.4 (2.2)	134.8 (2.2)	133.1 (2.3)	0.27
SAT (mL)	335.3 (4.9)	326.7(4.7)	332.8 (4.6)	337.5 (4.7)	344.7 (4.9)	0.07
IMAT (mL)	2.3 (0.06)	2.31 (0.05)	2.34 (0.05)	2.45 (0.05)	2.40 (0.06)	0.16
Anthropometry						
BMI (kg/m^2)	30.2 (0.2)	29.8 (0.2)	30.2 (0.2)	30.5 (0.2)	30.8 (0.2)	0.004
Weight (lb)	192.3 (1.3)	190.0 (1.2)	193.0 (1.2)	194.8 (1.2)	197.3 (1.3)	<0.001
WC (cm)	94.5 (0.5)	93.8 (0.5)	94.5 (0.4)	95.1 (0.5)	95.4 (0.5)	0.05
25-year change*						
BMI change (kg/m^2)	5.6 (0.2)	5.8 (0.2)	5.9 (0.2)	5.7 (0.2)	5.8 (0.2)	0.5
Weight gain (lb)	35.3 (1.4)	36.9 (1.3)	37.9 (1.3)	36.5 (1.3)	37.5 (1.4)	0.4
WC change (cm)	16.7 (0.5)	17.3 (0.5)	17.2 (0.5)	16.9 (0.5)	16.7 (0.5)	0.8

Models adjusted for age, sex, race, field center, education, height, smoking, alcohol consumption status, physical activity, energy intake, and HEI2015 score

*Change in BMI, weight, and WC were also adjusted for baseline BMI, weight, or WC as appropriate.

CARDIA = Coronary Artery Risk Development in Young Adults; VAT=visceral adipose tissue; SAT=subcutaneous adipose tissue; IMAT= intermuscular adipose tissue; HEI2015 = healthy eating index-2015; BMI = body mass index; WC=waist circumference

Supplementary Table 5. Risk of incident obesity associated with artificial sweetener and diet beverage intakes [hazard ratios (95% CIs)] over a median 17.5-year period among CARDIA participants (N=2 745, n events=1 142).

ArtSw exposure	Categories of ArtSw exposure 1	2	3	4	5	*p* for trend
Total ArtSw	Ref	1.04 (0.87, 1.23)	1.24 (1.05, 1.46)	1.28 (1.07, 1.52)	1.78 (1.51, 2.09)	<0.001
Aspartame	Ref	1.05 (0.89, 1.24)	1.21 (1.02, 1.44)	1.17 (0.98, 1.41)	1.64 (1.39, 1.93)	<0.001
Sucralose	Ref	0.92 (0.78, 1.09)	1.01 (0.85, 1.19)	1.08 (0.91, 1.28)	1.18 (0.99, 1.40)	0.018
Diet soda	Ref	1.01 (0.85, 1.20)	1.17 (0.99, 1.39)	1.30 (1.10, 1.53)	1.57 (1.34, 1.85)	<0.001
Saccharin*	Ref	1.13 (1.06, 1.20)	1.19 (1.13,1.26)			0.13

Cox regression models adjusted for age, sex, race, field center, education, height, smoking, alcohol consumption status, physical activity, energy intake, and HEI2015 score

*Saccharin was analysed by tertiles due to the high number of individuals with no reported saccharin intake

CARDIA = Coronary Artery Risk Development in Young Adults; ArtSw=artificial sweetener; VAT=visceral adipose tissue; SAT=subcutaneous adipose tissue; IMAT= intermuscular adipose tissue; HEI2015 = healthy eating index-2015; BMI = body mass index; WC=waist circumference
